# "What families want - an assessment of family expectations in the ICU"

**DOI:** 10.1186/1755-7682-4-21

**Published:** 2011-06-22

**Authors:** Shahla Siddiqui, Farheen Sheikh, Rehana Kamal

**Affiliations:** 1 Department of Anaesthesia, Aga Khan University, Stadium Road, Karachi, Pakistan

## Abstract

**Introduction:**

Families of patients admitted in the intensive care units (ICUs) experience high levels of emotional stress. Access to information about patient's medical conditions and quality relationships with healthcare staff are high priority needs for these families and meeting these needs of the family members is a primary responsibility of ICU physicians and nurses.

**Methodology:**

Our objectives were to assess the expectations of ICU patients' families that can be fulfilled by physicians and nurses. The design was a descriptive, exploratory questionnaire based study over 6 months in the multidisciplinary ICU of a tertiary care hospital.

**Results:**

Of 205 interviews, the median age of the patient was 28 years. One hundred and nineteen (58%) were male and Eighty six (42%) patients were female. 163 (79.5%) of the relatives were Next of kin, and 133 (64.9%) were male members. Of the family members, 20 (9.8%) were spouses. One hundred and forty two (69.3%) belonged to Middle income group. Ninety nine (48.3%) were Graduates of high school or above. Relation to patient, sex of relative, DNR status of patient and age of relative were statistically significant to make a difference to the satisfaction score. The majority of the relatives reached a score of 22-25.

**Conclusion:**

We conclude that families of critically ill patients were generally satisfied with communication in the ICU; however, our limitations are the cohort in our urban based tertiary care hospital may not adequately represent the majority of our population which is poor and illiterate and many other factors such as misunderstanding of medical knowledge and a more patriarchal attitude of physicians may affect family needs and satisfaction scores.

## Introduction

Families of critical care patients experience high levels of emotional stress [1]. Access to information about patient's medical conditions and quality relationships with healthcare staff are high priority needs for these families and meeting these needs of the family members is a primary responsibility of intensive care unit (ICU) physicians and nurses [2]. It can also be an important criterion in assessment of quality of care in the ICU. In previously done surveys in the West the most pressing need of family members of patients in the intensive care unit is to receive clear, understandable, and honest information about the patient's condition [3]. Admission to the ICU often comes with no warning, throwing families into a whirlwind of uncertainty, shock, helplessness and confusion. In our culture, religion and family support is of undeniable value. However, in the experience of ICU physicians, families have expectations and needs from healthcare providers (physicians and nurses) which are commonly overlooked or become secondary to caring for the patient [4]. Little research has been done on interventions for families of critically ill patients and almost none has been done on how to improve communication between ICU healthcare team and patients' family members[5]. Early work with families in the ICU elicited their perceived needs. Soon after, Molter (1976) investigated in depth and identified five general needs categories: information, assurance, proximity, support and comfort. The survey was officially developed into the critical care Family Needs survey (CCFNI) [6,7].

There are very few health care insurance schemes in the developing world and most patients admitted have to self pay. Hardly any work has been done in this field in our part of the world looking at the needs and expectations of the families of critically ill patients. The socioeconomic implications have also not been explored, for instance, does the lack of finances affect the families' decisions and perceptions of the care being delivered? There are many unanswered questions which given our unique cultural, economic and religious background make extrapolating Western studies to our part of the world difficult [8]. We carried out a study in our multidisciplinary ICU (comprising of medical, surgical, obstetrics and neurosurgery patients) to assess the expectations of our ICU families from the healthcare providers.

## Methodology

Our objectives were 1) to determine the expectations of families of patients admitted in the ICU - that can be fulfilled by physicians and nurses and 2) to create an assessment tool (questionnaire) addressing the communication with patients' families. Formal permission was obtained from the Ethical Review Committee of the Institution (ERC). Our Design was a questionnaire based descriptive, exploratory, multiple case study. By using semi structured interviews the following needs were graded: information, assurance, proximity, support, comfort, trust, religiousness [9]. The questionnaire was pretested prior to starting formal testing. Ten sample questionnaires were used and amendments made. Sample and duration: Our sample was convenience samples of consecutive patients via a Questionnaire as shown in Table 1. Since we did not have any previous studies showing us a response rate or prevalence of various variables we could not calculate a sample size and relied on collecting data over a period of 6 months. This resulted in a sample size of 205 patients. Setting: Our study was conducted in the waiting area of our 12 bedded, open, multi- disciplinary ICU of a tertiary care unit at the Aga Khan University Hospital, Karachi. The healthcare providers included the Primary team admitting the patient, an ICU team comprising of an ICU consultant and several residents, bedside nurses and various technicians. Inclusion Criteria: Adult, immediate family members present at the bedside for more than 2 days. This is taken as half of average Length of stay of an ICU patient (i.e. 4 days) according to the current census of ICU admissions.

**Table 1:** Analysis of Parameter Estimates

Parameter	Estimates	Standard Error	Wald 95% confidence limits		Chi-square
Kin	0.007	0.03	-0.06	0.077	0.04

Head of family	-0.02	0.03	-0.08	0.039	0.45

ICU team	-0.03	0.02	-0.09	0.02	1.44

Gender(ICU)	0.03	0.02	-0.01	0.095	1.70

Sex(relative)	0.0007	0.03	-0.05	0.05	0.00

DNR	-0.02	0.20	-0.43	0.38	0.01

Status	-0.04	0.02	-0.09	0.01	2.09

ICU days	0.005	0.004	-0.002	0.01	1.64

Education	0.01	0.02	-0.02	0.06	0.52

Age of relative	0.0003	0.001	-0.002	0.002	0.05

Exclusion criteria: Family members less than 18 yrs of age. Data Collection: A research assistant was hired to recruit family members in a consecutive manner at the start of the study, using the inclusion criterion. Multiple family members were recruited per patient separately within 24 hours of admission at one time point. They were assigned a serial number and informed consent was obtained prior to entering the study.

Family members usually wait outside the ICU in the waiting room where they were recruited, however the actual tool (Additional File 1) was delivered in the privacy of a 'counseling room' located within the ICU suite. The interview was conducted in Urdu after obtaining consent, the native language of the region. It was stressed to them at the start that the interview is being conducted by impartial observers who are not responsible in the care of their particular patient. As shown in Appendix I: Data Collection form included the following:

1. Patient Characteristics - Clinical and epidemiological parameters to determine condition of patient e.g. ASA classification, physician's notes, etc.

2. Family members' Characteristics and relationship to patient.

3. Satisfaction scale items - 25 items related to perception of care for the patient and themselves by the doctors and nurses.

Data Analysis:

The data was analyzed using a constant comparative process [6,10-12] in which data collection and analysis occurs simultaneously. The questionnaire comprised of 25 questions with simple open ended answers. Parameters were coded and analyzed and compared to previous data. Data was entered on SPSS.

## Results

A total of 205 family members were interviewed. 1. Patient Characteristics: The median age of the patients was 28 years (range of 0-85 yrs). 65% of the patients spent 1-5 days in the ICU; 27% spent 6-9 days, 6% spent 10-15 days and 2% spent > 15 days. 58% pateitns were males and only 33% were heads of their households. The pre ICU condition of the patients as denoted by the American Society of Anaesthesiologists classification (ASA) was 16% ASA I, which means there were no co-morbid conditions; 17% were ASA II, which means controlled co-morbid diseases; 49% were ASA III, which means uncontrolled co-morbids and 18% were ASA IV which means their chances of mortality were > 80%. Only 1 patient (0.5%) was DNR (Do not resuscitate) status. 43% of the patients were admitted under an Internal medicine team whilst the rest were almost equally distributed between Surgery, Paediatrics and Neurosurgery. 2. Characteristics of family member interviewed: 80% of the interviewees were next of kin relatives with 46% being a blood relation, 10% were spouses and the rest were 'others'. Median age of the relative was 34 years (range 18-70) and 65% were males. 45% relatives were only Undergraduate passed (highschool), 24% were Graduates (college), 16% were Post graduate trained (technical or University training) and 8% were Professionals whilst 7% were uneducated. As far as their Economic conditions were concerned, the majority, 69% were middle income, with 27% being poor and the rest were wealthy (3%). 3. Satisfaction Scale Items: 25 questions asked relating to this. Most (38%) of the interviewees had one doctor taking care of their loved one, whilst 16% 'Did not know'. 59% of them were aware of a separate ICU team as well as a Primary team. 89% of them had met with their doctors, 10% had not and 15 'did not know'. With 65% of the relatives the meeting took place once a day, with 10% it was only twice a day and with 7% there were multiple meetings per day. There were no meetings held with 8% of the interviewees, 6% had the meeting on the second day and 3% had it on the third day of admission. When asked their preferences, 44% said they wld like to meet with their doctor twice a day, 26% were satisfied with one meeting and 29% wanted meetings 'multiple' times per day. The meetings took place in the meeting room in 46% of the time, in the ICU 39% of the time, in the waiting area outside the ICU 12% of the time and in the doctor's clinic 4% of the time. The majority (46%) of the meetings were 5-10 minutes long, 26% were < 5 minutes and 28% were > 10 minutes long. 92% of the interviewees felt they had a chance to ask all their questions during the meetings. 94% felt these answers were understandable. Half of the interviewees liked the interview area (50%). 98% saw their patients every day and 74% felt the length of time for seeing the patient was adequate. 89% felt that the nurses regularly updated them, 9% felt they did not and 2% felt that some did. Whilst 92% felt that the caregivers should offer support to them emotionally, 90% felt that the doctors were sympathetic, and 82% felt that they offered support. In 44% cases the 'bad news' was delivered to them by the Consultant; an equal number had good news delivered by the Consultant. The rest was by either the resident, nurse or others, and the majority of the interviewees (54%) felt that it they would like to hear bad news from Consultants. 62% people wanted to see the patient during unstable periods. An equal number (31 and 32%) found comfort in 'talking to the doctor' and 'praying' whilst 15% found it in 'being with the patient' and 21% in 'hearing good news'.

42% ranked 'decisive and strong' as the most important characteristic in their doctor and an equal number wanted them to be 'soft and sympathetic'; 15% felt they should be both. 59% relatives wanted all major decisions to be made by the doctor and 97% felt correct information was more important when compared with 'support and empathy'. 51% considered 'Leaving it up to God' more important than 'Doctors doing everything aggressively', 30% felt the latter and 19% felt both were important. The overall satisfaction goal score 23.57 +- 3.9 (SD). Each variable was taken as a predictor of family satisfaction using the results of the univariate Poisson regression model to compute the expected percentage change in the satisfaction score with each one unit decrease in the dependent variable. This statistical test was done using the SAS 6.12 (SAS Inc., Cary, NC) package. Table 1. *Analysis of parameter estimates*, shows the significance tests of the model parameters. As shown, the relationship and the sex of the next of kin, DNR status of the patient, and the age of the relative were positively associated with the satisfaction score.

## Conclusion

Communication in the ICU with family members remains a grey area with variations from person to person. Adequate and effective communication with family members is the key to substitute decision making, thereby protecting patient autonomy [13,14], however often in the busy pace of a critical care environment this communication is not a priority. Well meaning physicians and nursing staff can often neglect this area of care with the result that misunderstanding and frustration can brew leading to medico legal and other implications. The positive effect of family support on the outcome from serious illness that requires intensive care has been recognized by clinicians for decades. We have all seen that family visitation and an intensive care environment more similar to that of a general ward can benefit patients with psychosis related to intensive care. The severity of illness of the individual patient exerts a powerful stress on the family unit, but it has been difficult to measure this effect [15,16]. We devised our assessment tool (questionnaire) based on several parameters including patient factors, relatives factors and a satisfaction score. The results of our study has shown that our patient cohort belonged to the younger age group, with the median age being 28 years, average ICU stay was 1-5 days and the majority of the patients were quite ill (ASA III). Surprisingly only 0.5% were DNR at the time of testing. Most of the next of kin were a decade (34 years) older and male, with only a high school level education and belonged to the middle income economic status. The majority of them was well aware of the intensity of illness of their relative, aware of the structure of the ICU care teams and had met their doctors at least once a day for 5-10 minutes although they mostly wanted to meet twice a day. Of the satisfaction score items most relatives were happy with the information imparted to them and in the way and place it was imparted. They expected their doctors and nurses to offer support emotionally and liked them to be strong and yet sympathetic equally. They wanted the Consultants to play the major role in imparting good or bad news and valued time spent with the patient especially during unstable periods, talking to the doctor and praying. Surprisingly the majority wanted the doctors to make all major decisions regarding the patients and ranked correct information much higher than support and sympathy. They also preferred to be more accepting of unfavourable outcomes, when compared to the Western cohorts studied [17,18] preferring to leave it all to God rather than doctors pursuing heroic measures aggressively. The average satisfaction score reflects a moderately high level of satisfaction with age, male sex, next of kin relationship of the relative and DNR status of the patient only significantly related. This could reflect a greater level of satisfaction when the patient's code status had been decided already as this was usually after mutual discussion and prolonged clarifications. Clearly, as evident in most critical care literature, proper communication is the crux of ensuring satisfaction and understanding of the expectations of these very distressed individuals [16,19].

Families of critically ill patients were generally satisfied with communication in the ICU; however, our limitations are the cohort in our urban based tertiary care hospital may not adequately represent the majority of our population which is poor and illiterate and many other factors such as misunderstanding of medical knowledge and a more patriarchal attitude of physicians may affect family needs and satisfaction scores. We therefore need to expand our study to multiple centers around the country to get a more holistic picture whilst at the same time acknowledging the current status which points towards detailed and frequent communication and a crucial role of the Consultant in all major discussions with families [20,21].

## Competing interests

The authors declare that they have no competing interests.

## Authors' contributions

SS conceptualized and designed the study, conducted the study, wrote the manuscript and helped in data entry and analysis; FS carried out the questionnaire survey and recruiting patients as well as data collection and data entry; RK helped in editing the manuscript.

All authors have read and approved the final manuscript.

## Supplementary Material

Additional file 1 **APPENDIX 1**. Data collection Form. Click here for file
