# Supplementary material for: "What families want - an assessment of family expectations in the ICU"
Source: Int Arch Med. 2011 Jun 22;4:21. doi: 10.1186/1755-7682-4-21 (PMC3130654; doi:10.1186/1755-7682-4-21)
Supplement: Additional file 1 — APPENDIX 1. Data collection Form. [file 1755-7682-4-21-S1.DOCX]

**APPENDIX I.** QUESTIONNAIRE ON ‘**AN ASSESSMENT OF EXPECTATIONS OF FAMILIES OF PATIENTS ADMITTED IN THE INTENSIVE CARE UNIT’.**

SUBJECT RELATED QUESTIONS:

Name:

Serial #:

Relationship:

Next of kin: yes no

Age:

Sex: M F

Profession:

Level of education: uneducated student graduate post graduate

professional healthcare professional

Economic Status: Poor Middle Income Wealthy

PATIENT RELATED QUESTIONS:

*ASAI=no comorbid diseases

ASAII=well controlled comorbids

ASAIII=

poorly controlled

ASAIV=patient is moribund

Age of patient:

# of days in ICU:

Sex of patient: M F

Head of household: yes no

Pre ICU condition of patient: ASAI ASAII ASAIII ASAIV*

*(american society of anaesthesiology classes)*

Is patient DNR? (do not resuscitate): yes no

Primary team: surgery medicine pediatrics

EXPECTATIONS AND KNOWLEDGE OF FAMILY MEMBERS:

1. How many doctors are taking care of your patient?

One two three four >five don’t know

2. Are you aware of a separate ICU team? Yes No

3. Have you met any of the doctors? Yes No don’t know

4. How often do you expect to meet your patient’s doctor in a day?

Once twice multiple times

5. How often do you meet the doctor?

Once twice multiple times no meeting

6. Where do your meetings take place?

Meeting room ICU waiting area clinic

7. How long do the meetings take?

<5min 5-10min >10min

8. Do you get a chance to ask all your questions? Yes No

9. Are the answers understandable? Yes No

10. Do you like the waiting area? Yes No

11. Do you get the chance to see your patient everyday? Yes No

12. Is the length of time adequate? Yes No

13. Do the nurses update you frequently? Yes No

14. Do you think your doctors are sympathetic? Yes No   dont know

15. Do you expect the doctors and nurses to offer support to you? Yes No

16. Do they offer support? Yes No

17. Who tells you the bad news?

Nurse resident consultant ICU team none so far

18. Who tells you the good news?

Nurse resident consultant ICU team

19. How would you like to hear the bad news?

From nurse resident consultant ICU team anyone

20. Are you allowed to see the patient during unstable periods? Yes No

21. What gives you most comfort?

Talking to doctor to be with patient praying good news

22. Which of the following would you rank as most important in your doctor?

Decisive & strong Soft & sympathetic both

23. Who should make major decisions about your patient?

You doctor doctor, with consent

24. What do you rank as more important?

Correct information support & empathy

25. What do you rank as more important?

Leaving it up to Allah doctors doing everything aggressively both
